# Tea Tree Oil Nanoemulsion-Based Hydrogel Vehicle for Enhancing Topical Delivery of Neomycin

**DOI:** 10.3390/life12071011

**Published:** 2022-07-07

**Authors:** Heba S. Elsewedy, Tamer M. Shehata, Wafaa E. Soliman

**Affiliations:** 1 Department of Pharmaceutical Sciences, College of Clinical Pharmacy, King Faisal University, Alhofuf 36362, Al-Ahsa, Saudi Arabia; tshehata@kfu.edu.sa; 2 Department of Pharmaceutics and Industrial Pharmacy, Faculty of Pharmacy, Zagazig University, Zagazig 44519, Egypt; 3 Department of Biomedical Sciences, College of Clinical Pharmacy, King Faisal University, Alhofuf 36362, Al-Ahsa, Saudi Arabia; weahmed@kfu.edu.sa; 4 Department of Microbiology and Immunology, Faculty of Pharmacy, Delta University for Science and Technology, Gamasa, Mansoura 11152, Egypt

**Keywords:** nanoemulsion, tea tree oil, neomycin, optimization, antibacterial, topical delivery

## Abstract

The present investigation aims to improve the antimicrobial influence of certain antibacterial drugs, namely, neomycin (NEO), exploiting the benefits of natural oils such as tea tree oil (TTO). Therefore, a distinctive nanolipid formulation, namely, a nanoemulsion (NE), was developed using a Central Composite Factorial Design (CCD) approach depending on the amount of TTO and tween 80 as surfactant. The optimized NEO-NE formula exhibiting minimum globular size and maximum in vitro release was selected. For efficient topical delivery, NEO-NE was incorporated into a pre-formulated hydrogel. The developed NEO-NE-hydrogel was characterized by its physical characteristics such as pH, viscosity, and spreadability. Next, it was tested for stability under different conditions for 3 months. Ultimately, an irritation test was conducted followed by an antibacterial examination. The preparation demonstrated acceptable properties to be successfully applied topically. It showed non-significant changes in stability in both conditions up to 3 months storage when compared to a fresh preparation. It exhibited no irritation when applied on hairless animal skin. Finally, TTO revealed a good inhibition for the bacterial growth that could improve the influence of NEO antibacterial activity, indicating the efficiency of NE containing NEO prepared with TTO to be a promising antibacterial nanocarrier.

## 1. Introduction

Skin is one of the mechanical defense systems in the human body that acts as a barrier against pathogen invasion [1]. However, it can be exposed to microbial infections that require certain treatments via topical application. The strategy of applying drugs over the skin and providing their effectiveness directly to the target site is termed a topical drug delivery system [2]. Topical delivery is a more desirable and convenient strategy than other routes of administration owing to its great advantages [3], since it can overcome the first pass mechanism and the problems associated with swallowing [4]. Topical drug delivery systems can be used for treating a wide variety of disorders where they are available as analgesic [5], antifungal [6], anti-inflammatory [7], anticancer [8], antioxidant [9] and antibacterial formulations [10]. Ointments and creams are different conventional dosage forms that are broadly used topically; however, certain problems could limit their formulation. Mostly, inadequate drug loading capacity, poor spreadability, and certain stability problems are the most challenging complications. Therefore, it was necessary to overcome these weaknesses to reach a more comfortable and reproducible activity [11]. 

In light of that, an advanced approach termed nanotechnology has critically attracted attention. Nanotechnology is the science of developing different nanosystems with a nanoscale range of size, hiding the unrequired properties of active constituents and maximizing their therapeutic actions [12]. Nanosystems are formed by nanocarriers carrying the active moiety of the drug. These nanocarriers are numerous, such as liposome, ethosome, niosome, nanoparticles, and nanoemulsion [13]. 

Nanoemulsion (NE) is one of the currently settled nanocarriers that are colloidal systems of tiny globular size and, consequently, large surface area, which can enhance drug absorption and bioavailability as well [14]. Additionally, NE can offer controlled drug release and protect the formulation against degradation [15]. NE can be used for delivering drugs via different ways of drug administration, oral [16], parenteral [17], transdermal [18], and topical routes [19]. Though, for topical medication delivery, it is more appropriate for the drug to be incorporated into a more viscous preparation such as hydrogel base providing the NE-hydrogel base formulation.

From the extensive spread of skin disorders, the prevalence of bacterial infections has appeared widely and should be handled wisely using antibacterial agents named antibiotics [20]. Neomycin sulfate (NEO) is one of the antibiotics that has revealed a broad-spectrum activity against Gram-positive and Gram-negative bacterial strains [21]. It is a 2-deoxystreptamine-containing aminoglycoside antibiotic exhibiting nephrotoxicity and ototoxicity problems upon long-term treatment, which explains its limited therapeutic range [22]. Despite that, its use was approved by the United States Food and Drug Administration [23]. Meanwhile, it was reported that the toxicity of the antibacterial agents is greatly minimized upon topical application [24,25]. Therefore, it was recommended to develop a topical formulation incorporating NEO rather than other routes. However, NEO was discovered long ago; thus, it could exhibit some kind of bacterial resistance [26]. Several investigations were explored in order to overcome bacterial resistance against NEO by producing a new derivative and applying structural modification [27,28]. Another strategy focused on combining NEO with other antibacterial agents that provided a superior influence than each one alone and helped in reducing the burden of bacteria, which is renowned as a combination therapy [29]. Given that there is a lack in the development of new antibiotics to face antimicrobial resistance, a combination therapy was an alternative way of choice for managing such complications that threaten the medicinal field [30].

The best category to be combined with these antibiotics is natural products owing to their excellent efficacy and safety [7]. Tea tree oil (TTO) is an essential oil of the Australian native plant *Melaleuca alternifolia*, a well-known genus derived from the Myrtaceae family [31]. With regard to its origin, it is well-known as melaleuca oil. TTO showed a broad-spectrum activity, namely, antiprotozoal, antifungal, and antiviral effects [32]. In addition to its antibacterial influence, which can be attributed to its cyclic monoterpenes structure from which terpoen-4-ol is responsible for such behavior [33], it also exhibits antioxidant and anticancer activity, which has formerly been proven, in addition to its established antiseptic and disinfectant influence [34].

In light of the previous facts, our target in the study has been raised. It is an attempt to formulate NE using TTO containing NEO. As far as we know, this is the first combination of TTO and NEO into a nanoemulsion formulation intended for topical application. CCD strategy as a tool for quality by design approach was run employing a 2^2^ full factorial design to obtain a high-quality product and selected the optimized NE formulation. The optimized formula was characterized, incorporated into a hydrogel base, and inspected for its antibacterial activity.

## 2. Materials and Methods

### 2.1. Material

Neomycin was obtained from (Sisco Research Laboratories Pvt. Ltd., Taloja, Maharashtra, India). Tea tree oil was acquired from NOW^®^ Essential Oils (NOW Foods, Bloomingdale, IL, USA). Diethylene Glycol Monoethyl Ether (Transcutol^®^ P) was bought from Gattefosse SAS (Saint-priest Cedex-France). Tween 80 and Carboxymethylcellulose Sodium (NaCMC) were purchased from Sigma-Aldrich Co. (St Louis, MO, USA). Distearoyl phosphatidylethanolamine-N-[methoxy poly (ethylene glycol)-2000] (PEG-DSPE) was bought from Lipoid LLC., (Newark, NJ, USA). All other chemicals were of the finest grade available.

### 2.2. Designing the Experiment

QbD approach was exploited to maximize the desirable characteristics of the formulations via implementing CCD in which two factors, 2 level (2^2^) factorial design was constructed. In that design, two independent factors were selected, amount of TTO and tween 80 with symbols, A and B, respectively. They were inspected at two levels to show their influence on two different dependent responses, namely, globule size (Y_1_) and in vitro release (Y_2_). The strategy was implemented using Design-Expert version 12.0 software (Stat-Ease, Minneapolis, MN, USA) since it helps in data interpretation via analyzing the results using the Analysis of variance (ANOVA) test. The data were illustrated further by constructing certain modeling graphs such as a 3D response surface plot, one-factor graph, and perturbation plot. The linearity between the actual and observed response could be demonstrated by predicted versus actual plot. Moreover, mathematical polynomial equations provided by the design could as well establish the influence of the nominated independent factors on the studied response [35]. 

### 2.3. Development of NEO-NE

Different NE formulations were prepared using TTO and including NEO; a method lately reported by Shehata et al. was followed and data were displayed in Table 1 [7]. Fundamentally, the aqueous phase was prepared by dissolving 50 mg of NEO in distilled water containing a specified amount of tween 80. On the other side, 0.5 g of transcutol^®^ P and 50 mg of PEG-DSPE were added to a quantified amount of TTO to provide an oily phase. Together, the two phases were mixed for 10 min at 15,000 rpm using a high shear homogenizer (T 25 digital Ultra-Turrax, IKA, Staufen, Germany) after adjusting the volume to 10 mL with distilled water. The formed NE was exposed to sonication for 30 s in order to obtain a suitable globule size using probe sonicator (XL-2000, Qsonica, Newtown, CT, USA).

### 2.4. Characterization of Developed NE

#### Globule Size and Polydispersity Index (PDI) Determination

One of the essential parameters to be evaluated in NE preparation is their globule size along with the relative size distribution. For that, Zetasizer apparatus (Malvern Instruments Ltd., Worcestershire, UK) was used for determining the globule size and the corresponding PDI of the preparation. Briefly, about 5 µL of each NE was added to 3 mL distilled water in a disposable cuvette and measured at 25 °C [36].

### 2.5. In Vitro Study

To detect the percentage of NEO released from the preparation, in vitro release study was conducted using the ERWEKA dissolution system (ERWEKA, GmbH, Heusenstamm, Germany) as mentioned previously by Almostafa et al. [10]. Briefly, 1 mL of NEO-NE sample was added into a glass tube hung into the apparatus and closed from one side with a cellophane membrane (MWCO 2000–15,000). The tubes were suspended into the acceptor vehicle composed of 500 mL phosphate buffer pH 5.5 and kept at 32 °C to mimic the skin condition. The system was operated and tubes were allowed, rotating at 50 rpm. Samples of 3 mL were withdrawn from the media at specified time up to 3 h and replaced with the same volume of fresh media. The withdrawn sample was analyzed at ƛ_max_ 277 nm using UV. Spectrophotometer (JENWAY 6305, Bibby Scientific Ltd., Staffs, UK). Experiment was performed three times for each sample. 

### 2.6. Zeta Potential

The optimized NEO-NE was evaluated for its surface charge by conducting zeta potential measurements using Zetasizer apparatus (Malvern Instruments Ltd., Worcestershire, UK). A special electrophoretic cuvette was used at which 5 µL of the sample was diluted with distilled water and checked for their electrophoretic mobility at 25 °C [37].

### 2.7. Development of NEO-NE-Based Hydrogel

Following validation of the optimization, the optimized NEO-NE formulation was loaded into a pre-formulated hydrogel base in order to facilitate the formulation topical application over the skin. Then, 4% NaCMC hydrogel was prepared simply by dispersing the gelling agent over 10 mL distilled water and keep stirring using magnetic stirrer (Jeio Tech TM-14SB, Medline Scientific, Oxfordshire, UK) until the homogenous NaCMC hydrogel base was obtained. The optimized NEO-NE formulation was added to the hydrogel base and mixed continuously for 5 min using a mixer (Heidolph RZR 1, Heidolph Instruments, Schwabach, Germany) in receipt of consistent NEO-NE-based hydrogel formulation [38]. 

### 2.8. Characterizing the Developed NEO-NE-Based Hydrogel

#### 2.8.1. Visual Examination

Visual inspection of the developed formula is very important, to follow up on the state of the preparation. Therefore, the developed NEO-NE-based hydrogel formulation was visually observed for its physical characteristics such as appearance, color, and homogeneity.

#### 2.8.2. pH Measurement

In order to avoid any probable irritation that could happen due to variation in pH value between the skin and the applied formula, this measurement was performed. pH value was determined using a standardized pH meter (MW802, Milwaukee Instruments, Szeged, Hungary) [39].

#### 2.8.3. Viscosity

Viscosity of the topical formulation is essential in evaluating the preparation. Therefore, appropriate viscosity evaluates the run-off of the formulation as it is better to stay adhered to the affected area for a longer time [40]. The viscosity of the examined NEO-NE-based hydrogel formulation was measured utilizing Brookfield viscometer (DV-II+ Pro, USA) using spindle 63 and worked at 25 °C [41].

#### 2.8.4. Spreadability

Spreadability is a critical parameter that has to be validated in any topical formulations as it greatly affects their viscosity. Proper spreadability gives an assumption about the formulation that would spread easily and evenly over the skin. It was conducted by holding a certain amount of the examined formulation in between two slides made of glass and of about (25 cm × 25 cm). Specific load, usually 500 g was added over the slides for 1 min. The spreadability is calculated by measuring the diameter of the spreading formulation [42].

### 2.9. Scanning Electron Microscopy (SEM)

Morphology of the fabricated NEO-NE-hydrogel formula could be estimated by a microscopic technique using scanning electron microscopy (SEM), (JSM-6390LA, JEOL, Tokyo, Japan). Typically, a sample of the formulation was added on slabs, shielded with gold using a sputter coater, and scanned. Then, the morphology was identified under a lower vacuum at an acceleration voltage of 10 kV using different magnifications [43].

### 2.10. In Vitro Release of NEO from Different Developed Formulations

The same method mentioned in Section 2.5 was followed to detect the in vitro release of NEO from the developed NEO-NE based hydrogel compared to the optimized NEO-NE formula.

### 2.11. Kinetic Study

Kinetic studies explain the mechanism by which the drug could be released from the developed formulation. It could take place by one of the kinetic modeling systems, namely, zero-order reaction, first-order, Higuchi, and Korsmeyer–Peppas modeling. Each mechanism illustrates a relation between drug concentration and the time, in a special way to provide the most fitted model with the highest correlation value R^2^. Meanwhile, zero-order kinetics demonstrates a relationship between the drug concentrations against time, while first-order kinetics clarifies the relation between Log concentrations against the time. Regarding the Higuchi equation, it illustrated the relationship between drug concentrations against the square root of time (t^0.5^). However, if the relation was among Log concentration against Log time, the model seemed to obey the Korsmeyer–Peppas equation [44].

### 2.12. Stability Test

The developed NEO-NE-based hydrogel preparation was checked for its capability to stay unchanged upon storage in different conditions and for a definite period of time. The study was performed according to the guidelines of the International Conference on Harmonization (ICH) to evaluate different criteria of the formulation such as pH, viscosity, spreadability, and in vitro drug release. The study was conducted after storing the formulation at 4 ± 1 °C and at 25 ± 1 °C for a period of 1 and 3 months [45].

### 2.13. Animal 

#### 2.13.1. Animals

Male Wister rats were required for the present investigation from the Experimental Animal Research Center at King Saud University, Riyadh, KSA; with an average weight of 220–250 g. The rats were housed in an appropriate environmental condition with a 12 h dark/light cycle and free access to water and food.

#### 2.13.2. Statement of Animal Ethics

Handling of animals and the entire in vivo experiments implemented were performed in accordance with the regulations of ethical conduct for animal use at King Faisal University. The protocol of the experiment was issued by the Research Ethics Committee (REC) of King Faisal University approval number (KFU-REC/2022-May–ETHICS17). 

#### 2.13.3. Skin Irritation Test

The study provides an indication about the safety of the formulation. Primarily, one day before proceeding with the test, the hair from the dorsal part of the animal was shaved using clippers. The inspected formulation was uniformly distributed over the shaved area. Rats were kept under observation for 7 days following topical application of the formulation. Rats were checked for any abnormal signs such as irritation, edema, or erythema (redness). The reactions were determined by applying a sensitivity scale ranging as 0, 1, 2, or 3, which represents no reaction, minor, moderate, and severe erythema with or without edema, respectively [46].

### 2.14. Microbiological Study

A microbiological examination was conducted to determine the antibacterial activity of the NEO-NE-based hydrogel using the disk diffusion method. The study was carried out using different bacterial strains provided by the American Type Culture Collection (ATCC). Consequently, *Bacillus subtilus* (ATCC 10400), *Staphylococcus aureus* (ATCC 29213), *Klebsiella pneumoniae* (ATCC 10013), and *Escherichia coli* (*E. coli*) (ATCC 25922) were used in the study as representative microorganisms. Simply, a disk of about 12 mm diameter was made by a sterile cork borer in a Petri dish containing Moller–Hinton Agar, which is a media for bacterial culturing. Small amounts of the preparation were added in each disk in order to evaluate the inhibition zone made by NEO-NE-based hydrogel and blank NE, compared with NEO solution as a control. The experiments were carried out in triplicate for each bacterium with a mean value ± SD and the plates were incubated for 24 h at 37 °C. Afterward, the zone of inhibition was measured in each plate and recorded. 

### 2.15. Statistics

Results were regarded as significant when *p* value being < 0.05. All studies were performed in triplicate. To compare results between two groups, Student’s *t*-test was followed. Analysis of variance (ANOVA) followed by the least significant difference (LSD) as a post hoc test was conducted when comparing between groups. The analysis was carried out using SPSS statistics software, version 9 (IBM Corporation, Armonk, NY, USA).

## 3. Results

### 3.1. Model Fitting and Statistical Data Analysis

As displayed in Table 1, 11 experimental formulations were generated by CCD explaining the influence of TTO and tween 80 amounts as independent variables on the globule size and in vitro release responses. The formulations were divided into four factorial, four axial, and three central points. The constructed design offered a statistical analysis of the data, which is very necessary for model identification. It was observed that the quadratic model was the best fitting one for both responses as it possessed R^2^ values of 0.9957 and 0.9976 for Y_1_ and Y_2_, respectively. Regarding the model F-value, it was 229.65 and 415.48 for both Y_1_ and Y_2_, respectively, indicating that model is significant as seen in Table 2. Additionally, *p*-values of Y_1_ and Y_2_ are less than 0.05, signifying that the model terms A, B, and A^2^ are significant. The lack of Fit F-value is another parameter that should be non-significant in order to fit the model. In the current design, the Lack of Fit was 4.27 and 0.0278 with corresponding *p*-values of 0.1955 and 0.9920 for Y_1_ and Y_2_, respectively, denoting non-significant values. 

### 3.2. Characterization of Developed NEs

#### 3.2.1. Effect of Variables A and B on Y_1_

The globule size of the formulation is a valuable parameter to be evaluated [47]. As presented in Table 1, the globule size of all NE formulations appeared to range from (153 ± 2.0 to 334 ± 4.5). With careful observation of the results, it was perceptible that the larger globule size of NE was obtained upon using a higher amount of TTO due to an increase in the dispersed phase [48]. Contrariwise, a smaller globule size of NE was attained upon using a higher amount of tween 80 while keeping the TTO amount constant. This could be accredited to using a higher amount of surfactant would lower the interfacial tension at the interface of the NE and, consequently, alter the probability of forming aggregates, keeping the globules small [49]. In addition, a higher amount of surfactant helps in maintaining the kinetic stability of the NE. The effect of both independent variables A and B on the globule size can be further illustrated by the following mathematical equation.

Y_1_ = 224.789 + 69.3333 A − 18.3333 × B − 2.5 AB + 18.5263 A^2^ 3.4736 B^2^

**Figure 1:**
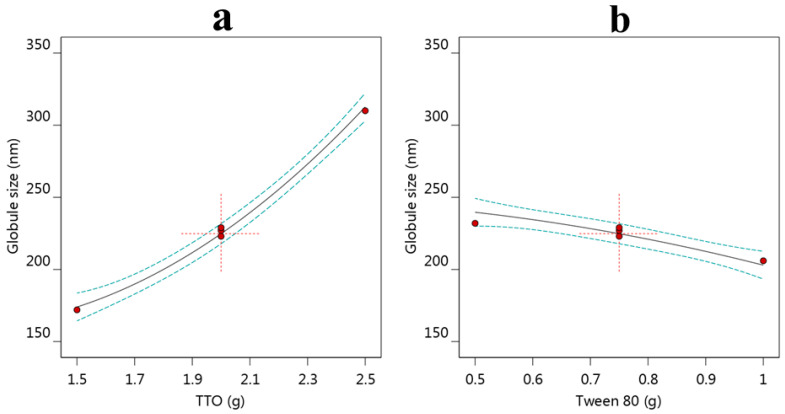
All factors plot representing (**a**) influence of variable A, and (**b**) influence of variable B, on the Y_1_ response.

From this equation, it is clear that the positive sign in front of factor (A) indicates its matching synergistic influence on the Y_1_ response; however, the negative sign appearing anterior to factor (B) implies an opposite influence on the same response. For extra clarification of the result, some graphical depictions were created such as an all-factor graph, as seen in Figure 1a, where the globule size was revealed to be increased by increasing the TTO amount, while in Figure 1b, it was noticed that there was a diminishing in the globule size by increasing the tween 80 amount. 

As well, it was listed in Table 3, the values of adjusted and predicted R^2^ for the Y_1_ response were 0.9913 and 0.9633, respectively. Both values seemed to be very near to each other, too close to 1, and the difference between them is less than 0.2, indicating that they were extremely correlated and fit the model. In addition, the value of adequate precision (45.1400) is presumed to be greater than four, which is desirable and could navigate the design.

#### 3.2.2. Effect of Variables A and B on Y_2_

The second dependent variable that was evaluated is the in vitro release experiment to outline the amount of NEO released from the developed NE formulation over a period of 12 h. It was obvious in Table 1 that the percentage of NEO released varied between 51 ± 3.0 and 84.8 ± 4.0%. These results disclosed that increasing the TTO amount from 2.5 to 3.5 g would result in a relative decrease in the formulation in vitro release pattern. The reason behind this returned to the globule size, whereas a higher amount of oil leads to a larger globule size with a corresponding small surface area that allowed the release of a small percentage of the drug from the formulation [49]. Contrariwise, the data showed that by applying the same amount of oil, the in vitro release would be enhanced by using a higher amount of surfactant. The developed mathematical equation could also verify the action of the independent variables A and B on the response of Y_2_. It was obvious from the equation that the negative impact of variable A on Y_2_ was confirmed with the negative sign; however, the direct positive effect was explained by the positive sign. The following is the mathematical equation generated by the design:

Y_2_ = 71.2474 − 14.2 A + 2.68333 B + 0.7 AB − 2.46842 A^2^ − 0.218421 B^2^

**Figure 2:**
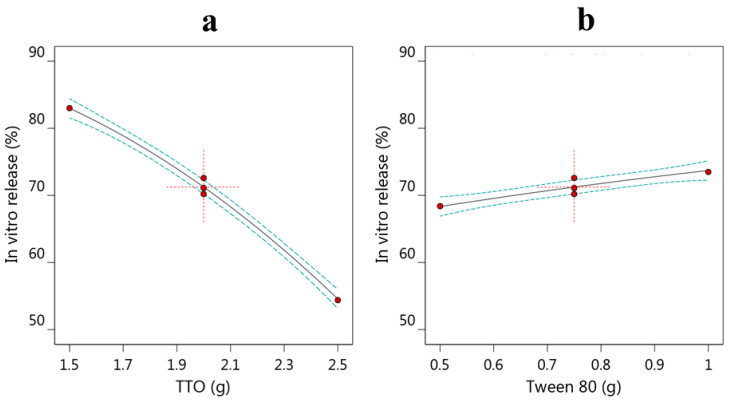
All factors plot representing (**a**) influence of variable A, and (**b**) influence of variable B, on the Y_2_ response.

Furthermore, the effect of variables A and B on Y_2_ was certified through particular model graphs designed by CCD, such as the all-factor graph, as exemplified in Figure 2. The influence of decreasing the in vitro release by increasing the TTO amount is displayed in Figure 2a, while Figure 2b displays the increase in that response upon increasing variable B. Moreover, the data shown in Table 3 present the value of the predicted and adjusted R^2^ (0.9949 and 0.9952), respectively, which were close to each other with a difference of less than 0.2, which means that they were in a reasonable agreement with each other. This would emphasize the linear correlation between actual and predicted values. In addition, the adequate precision value was 58.4163, indicating that such a model could navigate the design space. It was stated that an adequate precision ratio greater than four is desirable [50].

### 3.3. Optimization and Validation of Variables

**Figure 3:**
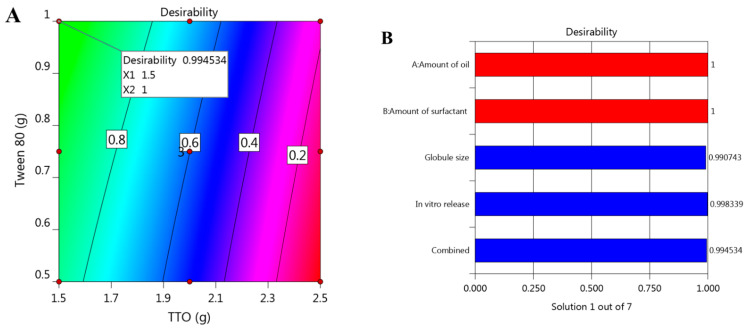
(**A**) 2D contour plot for desirability function displaying the effect of oil and surfactant amount on overall responses; and (**B**) desirability bar graph displaying the overall desirability of each response and combined optimization.

The process of optimizing the formulations depends on certain criteria related to the independent variables and their responses. Likewise, the optimization process depends on numerical optimization and the model graphs generated by the design software. The independent variables were adjusted to be in range; however, the dependent responses were oriented to provide the minimum globule size and the maximum in vitro release. Accordingly, a number of solutions were produced with corresponding desirability functions. The highest desirability value was (0.995) proposing the amount of TTO to be 1.5 g and that of tween 80 to be 1 g. Figure 3A shows a 3D plot for the overall desirability that investigated the desirable response, and Figure 3B illustrates the desirability for combined optimization. Based on the previous suggested data, a new formula that was expected to be the optimized one was formulated and the resultant responses were compared to the predicted values. Remarkably, the predicted and the observed values were very close to the extent that recommends the formula to be optimized, as displayed in Table 4.

### 3.4. Zeta Potential

**Figure 4:**
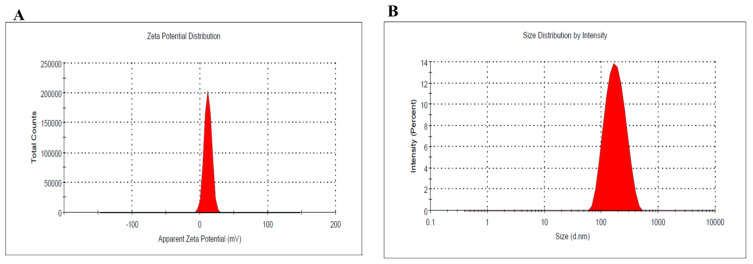
(**A**) Zeta potential, and (**B**) globule size and size distribution of the optimized NEO-NE.

Zeta potential is a very important parameter to be evaluated for detecting the stability of the formula. According to Figure 4A, the zeta potential of the optimized NEO-NE was determined and found to be 0.201, which leans towards neutral. The rationale behind this retuned to modifying the surface of the NE preparation with a hydrophilic polymer such as PEG-DSPE. This modification caused the overall charges on the surface of the formulation to be reduced since PEG helps to increase the hydrophilicity of the formula and, subsequently, prevent the clumping of their globules, providing higher stability [51]. This came in accordance with numerous investigations that supported the electrical neutrality of PEG and its role in improving the stability of the nanosystems [52,53]. Therefore, our findings propose the physical stability of the NEO-NE formulation that could be confirmed through stability testing. Furthermore, the globule size of the optimized NEO-NE was detected to be 161.3 ± 3.2 with relative PDI 0.145, as seen in Figure 4B. 

### 3.5. Characterizing the Developed NEO-NE-Based Hydrogel

Based on the former data obtained, and for achieving a more efficient topical preparation, the optimized NEO-NE formulation was incorporated into a pre-prepared hydrogel formulation. The NE and hydrogel base were mixed together via gentle stirring until NEO-NE-hydrogel was attained and kept for the next studies. 

#### 3.5.1. Visual Examination

The prepared NEO-NE-hydrogel was evaluated visually for its final appearance and found to be consistent and homogenous without phase separation.

#### 3.5.2. pH Measurement

The pH of the developed hydrogel base formulation was 6.34 ± 0.18, which seemed to be in great similarity with human skin pH, which guarantees its safety upon topical application [54]. 

#### 3.5.3. Viscosity

NEO-NE-hydrogel preparation was examined for the viscosity parameter. It was 14,680 ± 1045.9 cP. The result was satisfactory in regard to topical formulations and seemed to be consistent and would not run off easily when applied over the skin [55].

#### 3.5.4. Spreadability

Spreadability measurements were implemented to assess how easily the formulation would spread upon application. It is 55.7 ± 1.5 mm, which is adequate for any topical preparation [7].

### 3.6. SEM

**Figure 5:**
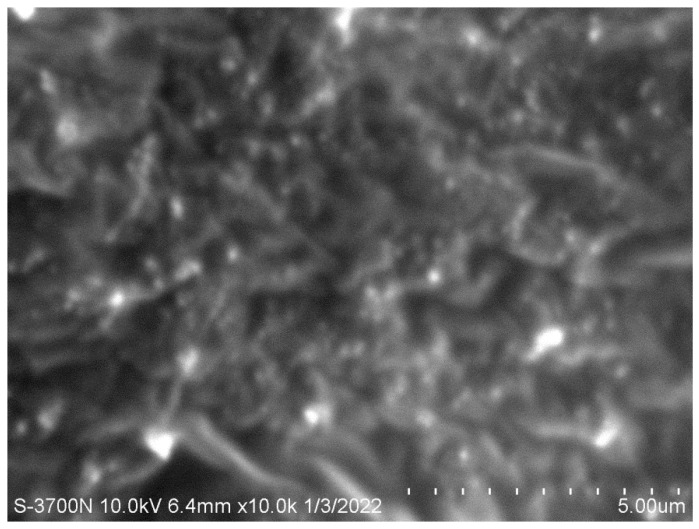
Scanning electron microscopy screening the morphology of developed NEO-NE-hydrogel.

The surface morphology and shape of the developed NEO-NE-hydrogel formulation were visualized utilizing SEM apparatus, as is apparent in Figure 5. It was distinguished from SEM analysis that the hydrogel base appeared as a network through which discrete spherical vesicles were dispersed without aggregation, suggesting the nanoemulsion preparation.

### 3.7. In Vitro Release of NEO from Different Developed Formulations

**Figure 6:**
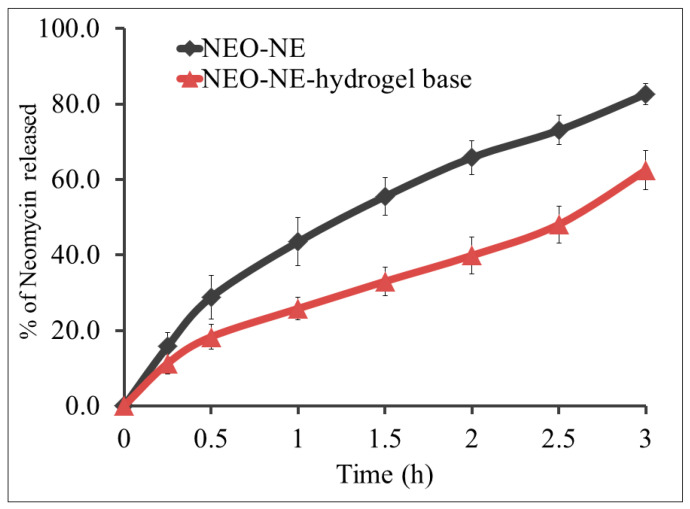
In vitro release of NEO from NEO-NE and NEO-NE-hydrogel in phosphate buffer pH 5.5 at 32 °C. Results are expressed as mean ± SD of three experiments.

The release of NEO from the prepared NE hydrogel formulation was investigated over 6 h; comparable to NEO released from NE itself, and the outline of the release was depicted in Figure 6. It was observed that the percentage of NEO released from NE was 82.6 ± 2.8%, which is greatly higher than that released from NE hydrogel (62.5 ± 5.1%). It is well known that the presence of a gelling agent in the formulation plays a key role in the viscosity of the formulation and the in vitro release as well [56]. Highly viscous formulations regularly exhibited a lower percentage of drug release [57]. It is highly noted that the NEO-NE-hydrogel formulation demonstrated higher viscosity when compared to the NEO-NE formulation as a result of integrating NaCMC. Interestingly, while NEO-NE revealed better in vitro release behavior, the NE-hydrogel formulation still being more recommended for topical application since it demonstrated good physical properties adequate for topical preparations [58].

### 3.8. Kinetic Study

**Figure 7:**
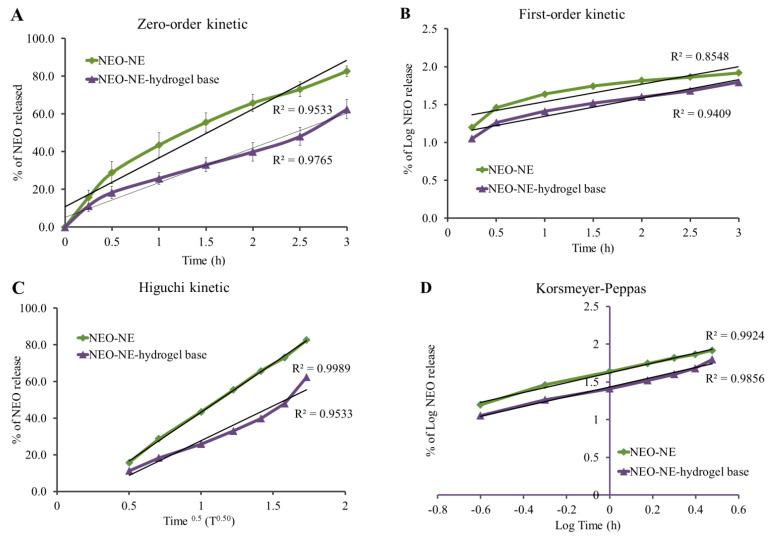
Percentage of NEO released from NE and NE-hydrogel base and their kinetic analysis relative to (**A**) Zero-order, (**B**) First-order, (**C**) Higuchi, and (**D**) Korsmeyer–Peppas kinetic model.

Different kinetic modeling was investigated in order to specify the mechanism by which NEO was released from NEO-NE and NEO-NE-hydrogel. In view of this, and as portrayed in Table 5 and Figure 7, it was clear that NEO release from NEO-NE formulation obeyed Higuchi kinetic modeling, since it provided the most linear correlation along with the highest value for R^2^, 0.9989. The release of the drug from the formulations is said to follow Higuchi kinetic modeling, once the diffusion of the drug is from lipid matrix type and under a controlled process [59,60]. On the other hand, the kinetic of NEO release from hydrogel base formulation is best explained by Korsmeyer–Peppas kinetic since it was a diffusion mechanism. This model provided the greatest R^2^ value (0.9856) when compared to the values of other models. It is well known that Korsmeyer–Peppas kinetic refers to the drug release from a polymer system such as a hydrogel base [61].

### 3.9. Stability Test

**Figure 8:**
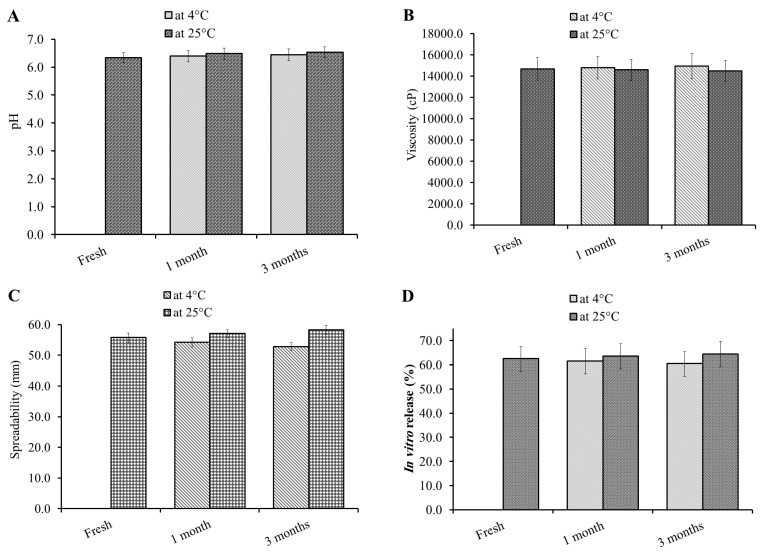
Stability profile of NEO-NE-hydrogel formulation following 1 and 3 months at 4 °C and 25 °C in terms of (**A**) pH; (**B**) % of drug content; (**C**) viscosity; (**D**) spreadability; and % of in vitro drug release compared to freshly prepared formulation.

The stability of the preparation is a highly required parameter to be investigated for determining the ideal storage condition and assuring the overall quality of the product [62]. The stability of NEO-NE-hydrogel formulation was conducted by storing at two different conditions for a period of 1 and 3 months, as shown in Figure 8. It was highly obvious that a non-significant difference was detected in the formulation upon storage at 4 ± 1 °C and at 25 ± 1 °C for the whole specified time of storage in terms of all examined parameters (*p* < 0.05). The previous outcomes could be returned to comprising PEG-DSPE in the formulation as a stabilizer.

### 3.10. In Vivo Study

#### In Vivo Skin Irritation Test

Rats treated with NEO-NE-hydrogel were checked for any irritation that could be noticed on their skin. Notably, no irritation, erythema, or edema was distinguished on the examined area throughout the whole investigation, which confirmed the safety of the formulations.

### 3.11. Microbiological Study

**Figure 9:**
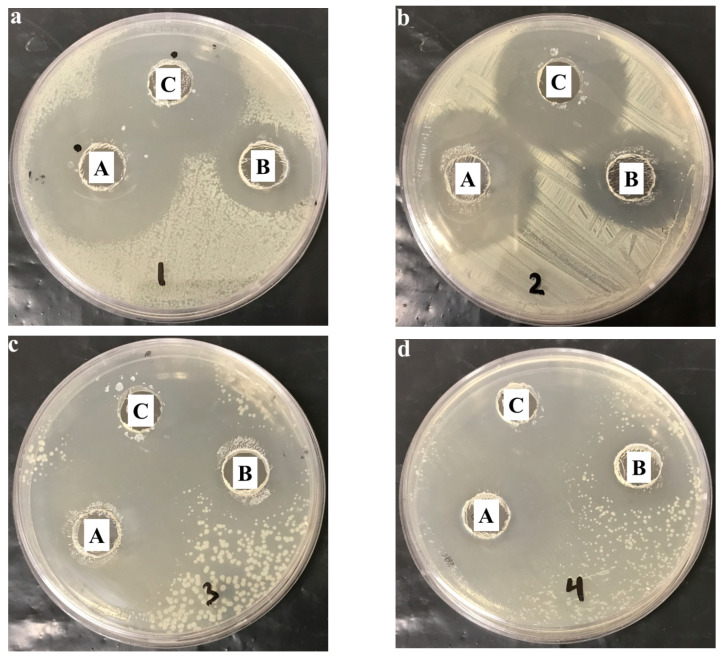
Inhibition zone diameter caused by investigated formulations: (A) NEEO-NE-hydrogel, (B) blank NE-hydrogel, and (C) NEO solution on different organisms: (**a**) *Bacillus subtilis*, (**b**) *Staphylococcus aureus*, (**c**) *klebsiella pneumonia*, and (**d**) *E-coli*.

The efficiency of NEO-NE-hydrogel formulation against certain Gram-positive and Gram-negative bacteria was evaluated by performing the disk diffusion method. The study was conducted using different formulations, namely, NEO-NE-hydrogel, blank NE-hydrogel, and NEO solution. It depends on measuring the inhibition zone diameter caused by the examined formulation against the bacteria, as shown in Figure 9 and Table 6. It was highly noted that there was a significant antibacterial effect detected by NEO-NE-hydrogel against the cultured bacteria, *Bacillus subtilis*, *Staphylococcus aureus*, *klebsiella pneumonia,* and *E-coli*. This is actually because the inhibition zone diameter caused by NEO-NE-hydrogel formulation was significantly higher than that caused by blank NE-hydrogel and NEO solution (*p* < 0.05). It was worth mentioning that the blank NEO-NE formulation containing TTO exhibited a considerable inhibition for the bacterial growth, which was definitely owed to the antibacterial behavior of the TTO. The antimicrobial activity of TTO against a wide range of bacteria was previously confirmed [33,63] and presumed to be principally due to its main content, terpinen-4-ol [64]. With regard to our findings, the greater antimicrobial activity revealed by the NEO-NE-hydrogel formula could be attributed to combining NEO and TTO, which resulted in the enhancement of the antibacterial activity of NEO.

## 4. Conclusions

In the present study, the effectiveness of neomycin as an antibacterial drug was enhanced via integrating it into a nanoemulsion prepared using Tea tree oil. A number of formulations were developed using Quality by Design technology based on tea tree oil and tween 80 amounts as independent variables. The influence of these factors on the globule size and in vitro release was investigated to determine the optimized formula. The optimized NEO-NE was incorporated into the gel formulation for convenient topical application. The developed hydrogel formulation showed good physical characteristics to be suitable for topical application. The formulation was stable over 3 months and did not show any sign of irritation. Tea tree oil prominently exhibited a considerable antibacterial activity that improves the action of NEO in inhibiting bacterial growth. Decisively, the nanoemulsion combined with a hydrogel base can be suggested as a topical drug delivery system.

## Figures and Tables

**Table 1:** Values of all independent factors and their detected dependent responses for different NE preparations.

Formula	Space Type	Independent Variables		Response Values		PDI
		A (g)	B (g)	Y_1_ (nm)	Y_2_ (%)	
F1	Factorial	1.5	1	153 ± 2.0	84.8 ± 4.0	0.29 ± 0.062
F2	Axial	1.5	0.75	172 ± 3.0	83.0 ± 3.6	0.32 ± 0.020
F3	Center	2	0.75	227 ± 4.4	70.2 ± 3.2	0.41 ± 0.011
F4	Factorial	1.5	0.5	190 ± 3.6	80.7 ± 4.1	0.34 ± 0.028
F5	Factorial	2.5	0.5	334 ± 4.5	51.0 ± 3.0	0.28 ± 0.029
F6	Center	2	0.75	229 ± 4.6	72.6 ± 3.2	0.39 ± 0.034
F7	Axial	2.5	0.75	310 ± 4.4	54.4 ± 3.7	0.32 ± 0.020
F8	Axial	2	1	206 ± 3.1	73.5 ± 3.1	0.28 ± 0.014
F9	Axial	2	0.5	232 ± 4.2	68.4 ± 2.9	0.30 ± 0.015
F10	Center	2	0.75	223 ± 3.0	71.1 ± 3.1	0.31 ± 0.014
F11	Factorial	2.5	1	287 ± 3.5	57.9 ± 2.8	0.26 ± 0.012

A: amount of TTO; B: amount of tween 80; Y_1_: globule size and Y_2_: In vitro release.

**Table 2:** Statistical analysis of responses.

Source	Y_1_		Y_2_	
	F-Value	*p*-Value	F-Value	*p*-Value
Model	229.65	<0.0001 *	415.48	<0.0001 *
A	1042.77	<0.0001 *	1975.05	<0.0001 *
B	72.91	0.0004 *	70.53	0.0004 *
AB	0.9038	0.3854	3.20	0.1337
A^2^	31.44	0.0025 *	25.20	0.0040 *
B^2^	1.11	0.3413	0.1973	0.6755
Lack of Fit	4.27	0.1955	0.0278	0.9920

A, amount of TTO (g); B, amount of tween 80 (g); Y_1_, globule size (nm); Y_2_, In vitro release (%); *, significant *p* < 0.05.

**Table 3:** Regression analysis and fit model summary statistics for the final suggested model that maximize the Adjusted and the Predicted R^2^.

Dependent Variable	Source	R^2^	Adjusted R^2^	Predicted R^2^	SD	Adequate Precision	Remark
Y_1_	Linear	0.9674	0.9593	0.9321	11.39	-	-
	2FI	0.9682	0.9546	0.8773	12.03	-	-
	Quadratic	0.9957	0.9913	0.9633	5.26	45.1400	Suggested
	Cubic	0.9984	0.9945	0.8636	4.19	-	-
Y_2_	Linear	0.9823	0.9779	0.9654	1.68	-	-
	2FI	0.9839	0.9769	0.9419	1.72	-	-
	Quadratic	0.9976	0.9952	0.9949	0.7827	58.4163	Suggested
	Cubic	0.9977	0.9922	0.9918	0.9966	-	-

**Table 4:** Predicted and observed values of the optimized NEO-NE formulation.

Independent Variable	Symbol	Criteria
Amount of TTO	A	In range
Amount of tween 80	B	In range
Dependent response	Predicted values	Observed values
Y_1_ (nm)	154.675 ± 5.25	161.3 ± 3.2
Y_2_ (%)	84.743 ± 0.78	82.63 ± 2.41

**Table 5:** Different kinetic modeling for demonstrating NEO release from NE and NE hydrogel-based formulations.

Kinetic Model	NEO-NE	NEO-NE-Hydrogel Base
Zero-order kinetic (R^2^)	0.9533	0.9765
First-order kinetic (R^2^)	0.8548	0.9409
Higuchi kinetic (R^2^)	0.9989	0.9533
Korsmeyer–Peppas kinetic (R^2^)	0.9924	0.9856

**Table 6:** Microbiological activity of examined formulations counters to different bacterial strains.

Inhibition Zone Diameter (cm)	Bacterial Type			
	*Bacillus subtilis*	*Staphylococcus aureus*	*klebsiella pneumoniae*	*E. coli*
NEO-NE-hydrogel	4.42 ± 0.13 * ^#^	4.26 ±0.11* ^#^	4.54 ± 0.11 * ^#^	4.38 ± 0.11 * ^#^
Blank NE-hydrogel	2.86 ± 0.12 *	3.09 ± 012 *	3.02 ±0.14 *	2.95 ± 0.13 *
NEO solution	4.14 ± 0.11 ^#^	4.01 ± 0.11 ^#^	4.29 ± 0.12 ^#^	4.12 ± 0.12 ^#^

Values are expressed as mean ± SD, n = 3. * (*p* < 0.05) compared to blank NE-hydrogel; and ^#^ (*p* < 0.05) compared to NEO solution.

## Data Availability

Not applicable.

## References

[1] Alkilani A.Z., McCrudden M.T.C., Donnelly R.F. (2015). Transdermal Drug Delivery: Innovative Pharmaceutical Developments Based on Disruption of the Barrier Properties of the Stratum Corneum. Pharmaceutics.

[2] Benson H.A., Grice J.E., Mohammed Y., Namjoshi S., Roberts M.S. (2019). Topical and Transdermal Drug Delivery: From Simple Potions to Smart Technologies. Curr. Drug Deliv..

[3] Rungseevijitprapa W., Yingngam B., Chaiyasut C. (2021). Improvement of Biophysical Skin Parameters of Topically Applied Fermented Soybean Extract-Loaded Niosomes with No Systemic Toxicity in Ovariectomized Rats. Pharmaceutics.

[4] Boztepe H., Ozdemir H., Karababa C., Yildiz O. (2014). Difficulties experienced during preparation and administration of oral drugs. Turk. Pediatri. Ars..

[5] Khullar R., Kumar D., Seth N., Saini S. (2011). Formulation and evaluation of mefenamic acid emulgel for topical delivery. Saudi Pharm. J..

[6] Kaur I.P., Kakkar S. (2010). Topical delivery of antifungal agents. Expert Opin. Drug Deliv..

[7] Shehata T.M., Elnahas H.M., Elsewedy H.S. (2022). Development, Characterization and Optimization of the Anti-Inflammatory Influence of Meloxicam Loaded into a Eucalyptus Oil-Based Nanoemulgel. Gels.

[8] Md S., Alhakamy N., Aldawsari H., Husain M., Khan N., Alfaleh M., Asfour H., Riadi Y., Bilgrami A., Akhter H. (2021). Plumbagin-Loaded Glycerosome Gel as Topical Delivery System for Skin Cancer Therapy. Polymers.

[9] Pinzaru I., Tanase A., Enatescu V., Coricovac D., Bociort F., Marcovici I., Watz C., Vlaia L., Soica C., Dehelean C. (2021). Proniosomal Gel for Topical Delivery of Rutin: Preparation, Physicochemical Characterization and In Vitro Toxicological Profile Using 3D Reconstructed Human Epidermis Tissue and 2D Cells. Antioxidants.

[10] Almostafa M.M., Elsewedy H.S., Shehata T.M., Soliman W.E. (2022). Novel Formulation of Fusidic Acid Incorporated into a Myrrh-Oil-Based Nanoemulgel for the Enhancement of Skin Bacterial Infection Treatment. Gels.

[11] Bashir M., Ahmad J., Asif M., Khan S.-U., Irfan M., Ibrahim A.Y., Asghar S., Khan I.U., Iqbal M.S., Haseeb A. (2021). Nanoemulgel, an Innovative Carrier for Diflunisal Topical Delivery with Profound Anti-Inflammatory Effect: In vitro and in vivo Evaluation. Int. J. Nanomed..

[12] Patra J.K., Das G., Fraceto L.F., Campos E.V.R., del Pilar Rodriguez-Torres M., Acosta-Torres L.S., Diaz-Torres L.A., Grillo R., Swamy M.K., Sharma S. (2018). Nano based drug delivery systems: Recent developments and future prospects. J. Nanobiotechnol..

[13] Wei Q.-Y., Xu Y.-M., Lau A.T.Y. (2020). Recent Progress of Nanocarrier-Based Therapy for Solid Malignancies. Cancers.

[14] Weiss J., Gaysinsky S., Davidson M., McClements J. (2009). Nanostructured encapsulation systems: Food antimicrobials. Global Issues in Food Science and Technology.

[15] Elsewedy H.S., Al-Dhubiab B.E., Mahdy M.A., Elnahas H.M. (2021). Basic Concepts of Nanoemulsion and its Potential application in Pharmaceutical, Cosmeceutical and Nutraceutical fields. Res. J. Pharm. Technol..

[16] Yen C.-C., Chen Y.-C., Wu M.-T., Wang C.-C., Wu Y.-T. (2018). Nanoemulsion as a strategy for improving the oral bioavailability and anti-inflammatory activity of andrographolide. Int. J. Nanomed..

[17] Harun S.N., Amin Nordin S., Abd Gani S.S., Shamsuddin A.F., Basri M., Bin Basri H. (2018). Development of nanoemulsion for efficient brain parenteral delivery of cefuroxime: Designs, characterizations, and pharmacokinetics. Int. J. Nanomed..

[18] Sharma A., Singh A.P., Harikumar S.L., Sl H. (2020). Development and optimization of nanoemulsion based gel for enhanced transdermal delivery of nitrendipine using box-behnken statistical design. Drug Dev. Ind. Pharm..

[19] Ansari M.N., Soliman G.A., Rehman N.U., Anwer K. (2022). Crisaborole Loaded Nanoemulsion Based Chitosan Gel: Formulation, Physicochemical Characterization and Wound Healing Studies. Gels.

[20] Bokhtia R.M., Girgis A.S., Ibrahim T.S., Rasslan F., Nossier E.S., Barghash R.F., Sakhuja R., Abdel-Aal E.H., Panda S.S., Al-Mahmoudy A.M.M. (2022). Synthesis, Antibacterial Evaluation, and Computational Studies of a Diverse Set of Linezolid Conjugates. Pharmaceuticals.

[21] Yu F., Zhang M., Sun J., Wang F., Li X., Liu Y., Wang Z., Zhao X., Li J., Chen J. (2022). Improved Neomycin Sulfate Potency in *Streptomyces fradiae* Using Atmospheric and Room Temperature Plasma (ARTP) Mutagenesis and Fermentation Medium Optimization. Microorganisms.

[22] A Manuel M., Kurtz I., Saiphoo C.S., Nedzelski J.M. (1979). Nephrotoxicity and ototoxicity following irrigation of wounds with neomycin. Can. J. Surg..

[23] Hosny K.M., Sindi A.M., Bakhaidar R.B., Zaki R.M., Abualsunun W.A., Alkhalidi H.M., Bahmdan R.H., Md S., Hassan A.H. (2020). Formulation and optimization of neomycin Sulfate–Thioctic acid loaded in a eucalyptus oil self-nanoemulsion to enhance the beneficial activity of the substances and limit the side effects associated with the treatment of hepatic coma. J. Drug Deliv. Sci. Technol..

[24] Bandyopadhyay D. (2021). Topical antibacterials in dermatology. Indian J. Dermatol..

[25] Wolverton S.E., Wu J. (2019). Comprehensive Dermatologic Drug Therapy.

[26] Bergen P.J., Landersdorfer C.B., Lee H.J., Li J., Nation R.L. (2012). ‘Old’ antibiotics for emerging multidrug-resistant bacteria. Curr. Opin. Infect. Dis..

[27] Zhang J., Keller K., Takemoto J.Y., Bensaci M., Litke A., Czyryca P.G., Chang C.-W.T. (2009). Synthesis and combinational antibacterial study of 5″-modified neomycin. J. Antibiot..

[28] Zhang J., Chiang F.-I., Wu L., Czyryca P.G., Li D., Chang C.-W.T. (2008). Surprising Alteration of Antibacterial Activity of 5′′-Modified Neomycin against Resistant Bacteria. J. Med. Chem..

[29] Blanchard C., Brooks L., Beckley A., Colquhoun J., Dewhurst S., Dunman P.M. (2016). Neomycin Sulfate Improves the Antimicrobial Activity of Mupirocin-Based Antibacterial Ointments. Antimicrob. Agents Chemother..

[30] Coates A., Hu Y., Holt J., Yeh P. (2020). Antibiotic combination therapy against resistant bacterial infections: Synergy, rejuvenation and resistance reduction. Expert Rev. Anti-Infective Ther..

[31] Yasin M., Younis A., Ramzan F., Javed T., Shabbir R., Noushahi H.A., Skalicky M., Ondrisik P., Brestic M., Hassan S. (2021). Extraction of Essential Oil from River Tea Tree (*Melaleuca bracteata* F. Muell.): Antioxidant and Antimicrobial Properties. Sustainability.

[32] Carson C.F., Hammer K.A., Riley T.V. (2006). *Melaleuca alternifolia* (Tea Tree) Oil: A Review of Antimicrobial and Other Medicinal Properties. Clin. Microbiol. Rev..

[33] Cox S.D., Mann C.M., Markham J.L., Gustafson J.E., Warmington J.R., Wyllie S.G. (2001). Determining the Antimicrobial Actions of Tea Tree Oil. Molecules.

[34] Yasin M., Younis A., Javed T., Akram A., Ahsan M., Shabbir R., Ali M.M., Tahir A., El-Ballat E.M., Sheteiwy M.S. (2021). River Tea Tree Oil: Composition, Antimicrobial and Antioxidant Activities, and Potential Applications in Agriculture. Plants.

[35] Shehata T.M., Khalil H.E., Elsewedy H.S., Soliman W.E. (2020). Myrrh essential oil-based nanolipid formulation for enhancement of the antihyperlipidemic effect of atorvastatin. J. Drug Deliv. Sci. Technol..

[36] Shehata T.M., Elsewedy H.S. (2022). Paclitaxel and Myrrh oil Combination Therapy for Enhancement of Cytotoxicity against Breast Cancer; QbD Approach. Processes.

[37] Haroun M., Elsewedy H.S., Shehata T.M., Tratrat C., Al Dhubiab B.E., Venugopala K.N., Almostafa M.M., Kochkar H., Elnahas H.M. (2022). Significant of injectable brucine PEGylated niosomes in treatment of MDA cancer cells. J. Drug Deliv. Sci. Technol..

[38] Abdallah M.H., Elsewedy H.S., AbuLila A.S., Almansour K., Unissa R., Elghamry H.A., Soliman M.S. (2021). Quality by Design for Optimizing a Novel Liposomal Jojoba Oil-Based Emulgel to Ameliorate the Anti-Inflammatory Effect of Brucine. Gels.

[39] Ayoub A.M., Ibrahim M.M., Abdallah M.H., Mahdy M.A. (2016). Sulpiride microemulsions as antipsychotic nasal drug delivery systems: In-vitro and pharmacodynamic study. J. Drug Deliv. Sci. Technol..

[40] Binder L., Mazál J., Petz R., Klang V., Valenta C. (2019). The role of viscosity on skin penetration from cellulose ether-based hydrogels. Ski. Res. Technol..

[41] Elsewedy H.S., Al Dhubiab B.E., Mahdy M.A., Elnahas H.M. (2020). Brucine PEGylated nanoemulsion: In vitro and in vivo evaluation. Coll. Surfaces A Physicochem. Eng. Asp..

[42] Abdallah M.H., Abu Lila A.S., Unissa R., Elsewedy H.S., Elghamry H.A., Soliman M.S. (2021). Brucine-Loaded Ethosomal Gel: Design, Optimization, and Anti-inflammatory Activity. AAPS PharmSciTech.

[43] Husseiny R.A., Abu Lila A.S., Abdallah M.H., Hamed E.E., El-Ghamry H.A. (2018). Design, in vitro/in vivo evaluation of meclizine HCl-loaded floating microspheres targeting pregnancy-related nausea and vomiting. J. Drug Deliv. Sci. Technol..

[44] Elsewedy H.S., Younis N.S., Shehata T.M., Mohamed M.E., Soliman W.E. (2021). Enhancement of Anti-Inflammatory Activity of Optimized Niosomal Colchicine Loaded into Jojoba Oil-Based Emulgel Using Response Surface Methodology. Gels.

[45] Abdelnabi D.M., Abdallah M.H., Elghamry H.A. (2019). Buspirone Hydrochloride Loaded In Situ Nanovesicular Gel as an Anxiolytic Nasal Drug Delivery System: In Vitro and Animal Studies. AAPS PharmSciTech.

[46] Soliman W.E., Shehata T.M., Mohamed M.E., Younis N.S., Elsewedy H.S. (2021). Enhancement of Curcumin Anti-Inflammatory Effect via Formulation into Myrrh Oil-Based Nanoemulgel. Polymers.

[47] Zainol S., Basri M., Bin Basri H., Shamsuddin A.F., Gani S.S.A., Karjiban R.A., Abdul-Malek E. (2012). Formulation Optimization of a Palm-Based Nanoemulsion System Containing Levodopa. Int. J. Mol. Sci..

[48] Jaiswal M., Dudhe R., Sharma P.K. (2015). Nanoemulsion: An advanced mode of drug delivery system. 3 Biotech.

[49] Sarheed O., Dibi M., Ramesh K.V.R.N.S. (2020). Studies on the Effect of Oil and Surfactant on the Formation of Alginate-Based O/W Lidocaine Nanocarriers Using Nanoemulsion Template. Pharmaceutics.

[50] Singh G., Ahuja N., Sharma P., Capalash N. (2009). Response surface methodology for the optimized production of an alkalophilic laccase from gamma-proteobacterium JB. BioResources.

[51] Wu B., Liu H., Chen G., Zhang Y., Ma Z. (2014). Effects of PEGylated lipid nanoparticles on the oral absorption of one BCS II drug: A mechanistic investigation. Int. J. Nanomed..

[52] Cieślak A., Wauthoz N., Orellana A.N., Lautram N., Béjaud J., Hureaux J., Lafleur M., Benoit J.-P., Salomon C.J., Bastiat G. (2017). Stealth nanocarriers based sterosomes using PEG post-insertion process. Eur. J. Pharm. Biopharm..

[53] Shi L., Zhang J., Zhao M., Tang S., Cheng X., Zhang W., Li W., Liu X., Peng H., Wang Q. (2021). Effects of polyethylene glycol on the surface of nanoparticles for targeted drug delivery. Nanoscale.

[54] Islam M.T., Rodríguez-Hornedo N., Ciotti S., Ackermann C. (2004). Rheological Characterization of Topical Carbomer Gels Neutralized to Different pH. Pharm. Res..

[55] Harish N., Prabhu P., Charyulu R., Gulzar M., Subrahmanyam E. (2009). Formulation and evaluation of *in situ* gels containing clotrimazole for oral candidiasis. Indian J. Pharm. Sci..

[56] Md S., Alhakamy N., Aldawsari H.M., Kotta S., Ahmad J., Akhter S., Alam S., Khan M.A., Awan Z., Sivakumar P.M. (2020). Improved Analgesic and Anti-Inflammatory Effect of Diclofenac Sodium by Topical Nanoemulgel: Formulation Development—In Vitro and In Vivo Studies. J. Chem..

[57] Yen W.F., Basri M., Ahmad M., Ismail M. (2015). Formulation and Evaluation of Galantamine Gel as Drug Reservoir in Transdermal Patch Delivery System. Sci. World J..

[58] Arora R., Aggarwal G., Harikumar S.L., Kaur K. (2014). Nanoemulsion Based Hydrogel for Enhanced Transdermal Delivery of Ketoprofen. Adv. Pharm..

[59] Pinheiro M., Ribeiro R., Vieira A., Andrade F., Reis S. (2016). Design of a nanostructured lipid carrier intended to improve the treatment of tuberculosis. Drug Des. Dev. Ther..

[60] Mircioiu C., Voicu V., Anuta V., Tudose A., Celia C., Paolino D., Fresta M., Sandulovici R., Mircioiu I. (2019). Mathematical Modeling of Release Kinetics from Supramolecular Drug Delivery Systems. Pharmaceutics.

[61] Sainy J., Atneriya U., Kori J.L., Maheshwari R. (2021). Development of an *Aloe vera*-based Emulgel for the Topical Delivery of Desoximetasone. Turk. J. Pharm. Sci..

[62] Osel N., Parfant T.P., Kristl A., Roškar R. (2021). Stability-Indicating Analytical Approach for Stability Evaluation of Lactoferrin. Pharmaceutics.

[63] Cox S.D., Mann C.M., Markham J.L., Bell H.C., Gustafson J.E., Warmington J.R., Wyllie S.G. (2001). The mode of antimicrobial action of the essential oil of Melaleuca alternifolia (tea tree oil). J. Appl. Microbiol..

[64] Southwell I., Hayes A., Markham J., Leach D. (1993). The search for optimally bioactive Australian tea tree oil. Acta Hortic..

